# Zika M Oligopeptide ZAMP Confers Cell Death-Promoting Capability to a Soluble Tumor-Associated Antigen through Caspase-3/7 Activation

**DOI:** 10.3390/ijms21249578

**Published:** 2020-12-16

**Authors:** Bénédicte Vanwalscappel, Juliano G. Haddad, Roba Almokdad, Jason Decotter, Gilles Gadea, Philippe Desprès

**Affiliations:** Processus Infectieux en Milieu Insulaire Tropical (PIMIT), Université de La Réunion, INSERM UMR 1187, CNRS 9192, IRD 249, Plateforme CYROI, 2 Rue Maxime Rivière, 97491 Sainte-Clotilde, La Réunion, France; benedicte.vanwalscappel@univ-reunion.fr (B.V.); juliano.haddad@univ-reunion.fr (J.G.H.); mokdadroba@gmail.com (R.A.); jason.decotter@univ-reunion.fr (J.D.); gilles.gadea@inserm.fr (G.G.)

**Keywords:** arbovirus, Zika virus, M protein, viral oligopeptide, apoptotic cell death, viral apoptosis inducer, caspase-3/7 activation, tumor cells, tumor-associated antigen, anticancer agent, anticancer virotherapy

## Abstract

Mosquito-borne Zika virus (ZIKV) is an emerging flavivirus of medical concern associated with neurological disorders. ZIKV utilizes apoptosis as a mechanism of cell killing. The structural M protein may play a role in flavivirus-induced apoptosis. The death-promoting capability of M has been restricted to an oligopeptide representing the residues M-32/40. Here, we evaluated the apoptosis inducing ability of the residues M-31/41 of ZIKV. The ZIKV M oligopeptide was associated to a soluble form of GFP (sGFP) and the resulting sGFP-M31/41 construct was assessed in Huh7 cells. Expression of sGFP-M31/41 can trigger apoptosis in Huh7 cells through caspase-3/7 activation. The translocation of sGFP-M31/41 in the endoplasmic reticulum was a prerequisite for apoptosis induction. The residues M-33/35/38 may play a critical role in the death-promoting activity of sGFP-M31/41. The effect of ZIKV M oligopeptide defined as ZAMP (for Zika Apoptosis M Peptide) on expression of a tumor-associated antigen was assayed on megakaryocyte-potentiating factor (MPF). Expression of MPF-ZAMP construct resulted in caspase-associated apoptosis activation in A549 and Huh7 cells. ZIKV has been proposed as an oncolytic virus for cancer therapy. The ability of the Zika M oligopeptide to confer death-promoting capability to MPF opens up attractive perspectives for ZAMP as an innovative anticancer agent.

## 1. Introduction

Zika virus is an emergent mosquito-borne, enveloped RNA virus belonging to the flavivirus genus of the *Flaviviridae* family. ZIKV is a neurotropic pathogen that mainly targets the central nervous system (CNS) [1], leading to several neurological diseases such as congenital neurological disorders and Guillain−Barré syndrome in adults [2,3]. ZIKV strains are clustered into two major lineages, the African and Asian genotypes [4], the latter being responsible for the current epidemics with a million cases of infection reported, in particular in South America. In addition to its conventional transmission by infected mosquito bite, human-to-human sexual or maternal-to-fetal transmission has been confirmed during the recent epidemics.

Like other flaviviruses such as dengue virus (DENV), yellow fever virus (YFV), and West Nile virus (WNV), ZIKV contains a single genomic RNA encoding a large polyprotein that is co- and post-translationally processed into three structural proteins C (capsid protein), prM (the intracellular precursor of the small membrane M protein), and E (envelope protein) followed by nonstructural proteins NS1 to NS5. The processing of prM in mature M protein (75 amino-acid residues) by the host furin/subtilisin protease family occurs in a post-Golgi compartment leading to the release of mature and infectious virus particles. The mature M protein consists of an ectodomain (hereafter referred to as ectoM) composed of amino acids M-1/40 followed by a transmembrane-anchoring region including two transmembrane domains (TMDs). It is of note that dengue M sequences are highly conserved among the four serotypes unlike other structural proteins.

It has recently been reported that expression of mature DENV M protein leads to inflammasome activation [5]. Historically, it had been demonstrated that expression of DENV ectoM conjugated to a reporter protein such as GFP can trigger apoptosis in human hepatoma cells [6]. The death-promoting activity is associated with a localization of DENV ectoM protein in a post-Golgi compartment [6]. Mutational analysis allowed the proapoptotic viral sequence to be restrained to the last C-terminal amino-acid residues M-32/40 of DENV ectoM which had been named ApoptoM [6]. Although the mechanism of ApoptoM-mediated cell death still needs to be better understood, apoptosis triggered by ApoptoM was associated with a mitochondrial dysfunction leading to activation of apoptosis executioner caspase-3 [7]. In the present study, we wondered whether the residues M-31/41 of epidemic Brazilian ZIKV strain BeH819015 could trigger apoptosis in human hepatoma and pulmonary adenocarcinoma cells. For this purpose, the ZIKV M oligopeptide representing the residues M-31/41 of BeH819015 was positioned at the C-terminus of reporter GFP and a tumor-associated antigen. We showed that recombinant proteins carrying the ZIKV residues M31/41 have the ability to trigger apoptosis in human cells through caspase-3/7 activation.

## 2. Results

### 2.1. Apoptosis-Inducing Ability of a Recombinant GFP Protein Carrying the ZIKV Residues M-31/41

**Figure 1:**
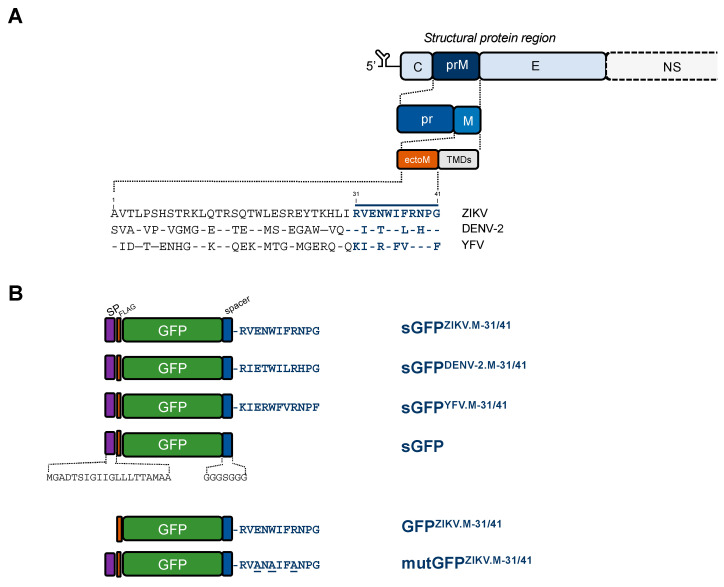
Schematic representation of the GFP-M oligopeptide constructs. In (**A**), a schematic representation of mature prM protein that is structured into a “pr” polypeptide followed by the residues M-1/41 which compose the M ectodomain and ending in a transmembrane anchoring region with two transmembrane domains (TMDs). The residues M-1/41 of epidemic Brazilian ZIKV strain BeH819015, epidemic La Reunion 2018 DENV-2 strain RUJul and YFV 17D vaccine strain are listed. The last C-terminal residues of flavivirus M ectodomain are underlined. In (**B**), the GFP constructs are preceded at the N-terminus by the signal peptide (SP) of ZIKV prM protein followed by a FLAG epitope. The soluble sGFP-M oligopeptide constructs are ended by a glycine−serine spacer followed by the residues M-31/41 of ZIKV, DENV-2 or YFV. The sGFP construct is restricted to a glycine−serine spacer at its C-terminus. The GFP^ZIKV.M-31/41^ construct is a mutant lacking in prM signal peptide. The mutGFP^ZIKV.M-31/41^ construct was made by replacing the residues M-E33/W35/R38 with alanine. The three Ala mutations in mutGFP^ZIKV.M-31/41^ are underlined.

We investigated whether the residues M-31/41 from epidemic ZIKV strain BeH819015 could trigger apoptosis as previously observed with DENV and YFV (Figure 1A). Consequently, we generated a soluble recombinant GFP (sGFP) protein in which the ZIKV M oligopeptide was added to its C-terminus (Figure 1B). The sGFP^ZIKV.M-31/41^ construct was preceded by the ZIKV prM signal peptide corresponding to the last amino-acid residues of BeH819015 C protein (Figure 1B). A same design of GFP-based constructs was applied for residues M-31/41 of DENV-2 and YFV to serve as positive controls. A sGFP construct with only the glycine−serine spacer at the C-terminus was used as a negative control (Figure 1B).

Given that human hepatoma cells were susceptible to death-promoting activity of DENV and YFV M ectodomains [6], transfection with plasmids expressing recombinant sGFP was performed in Huh7 cells. FACS analysis showed that GFP expression rate was similar between Huh7 cells expressing sGFP with or without M-31/41 sequence, indicating that the different GFP constructs were suitable for further characterization (Figure S1).

**Figure 2:**
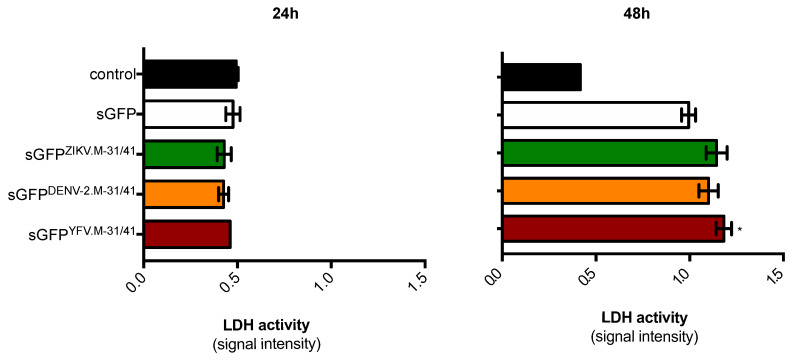
Effect of sGFP-M31/41 constructs on cell membrane integrity. Huh7 cells were transfected 24 h (left) or 48 h (right) with plasmids expressing sGFP or sGFP-M31/41 constructs, or mock-transfected cells (control). LDH activity was measured and cell membrane permeability was expressed as signal intensity (O.D.). The results are the mean (±SEM) of three (24 h) or six independent experiments (48 h). Statistical analysis for comparing sGFP-M constructs with sGFP was performed and noted (* *p* < 0.01); differences that were not statistically significant are omitted.

To determine whether expression of sGFP proteins carrying the different M oligopeptides has an effect on cell membrane integrity, Huh7 cells were transfected with vector plasmids expressing either sGFP^ZIKV.M-31/41^, sGFP^DENV-2.M-31/41^, or sGFP^YFV.M-31/41^ (Figure 2). The sGFP construct was used as a control. Cell membrane integrity was evaluated by measuring lactate dehydogenase (LDH) release (Figure 2). At 24 h post-transfection, no significant change in LDH activity was observed in transfected Huh7 cells whatever the recombinant GFP constructs tested. At 48 h post-transfection, LDH activity increased in Huh7 cells expressing the sGFP constructs as compared to control (Figure 2). Only the sGFP^YFV.M-31/41^ construct was slightly more cytotoxic compared to other sGFP constructs. From these results, it has been estimated that 24 h was the more appropriate time-point of transfection to assess the death-promoting capability of sGFP constructs with the flavivirus M oligopeptides in Huh7 cells.

**Figure 3:**
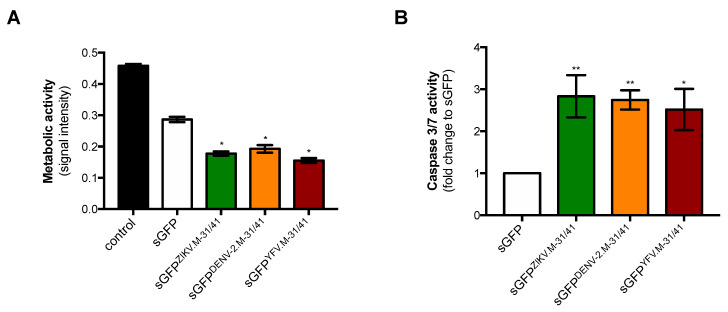
Expression of sGFP-M31/41 constructs affects cell viability. Huh7 cells were transfected 24 h with plasmids coding for sGFP^ZIKV.M-31/41^, sGFP^DENV-2.M-31/41,^ or sGFP^YFV.M-31/41^ or mock-transfected (control). The sGFP plasmid construct served as a control. In (**A**), the level of cell metabolic activity was measured using MTT assay and expressed as signal intensity (O.D.). The results are the mean (±SEM) of six independent assays. Statistical analysis for comparing sGFP-M constructs with sGFP was performed and noted (* *p* < 0.0001). In (**B**), Caspase 3/7 enzymatic activity was expressed as the fold change of caspase enzymatic activity in assay relative to sGFP. The results are the mean (±SEM) of six independent assays. Statistical analysis for comparing sGFP-M constructs with sGFP was performed and noted (** *p* < 0.001; * *p* < 0.01).

We evaluated whether sGFP^ZIKV.M-31/41^ expression had an effect on the metabolism of Huh7 cells by measuring MTT activity at 24 h post-transfection (Figure 3). As expected, both GFP^DENV-2.M31/41^ and GFP^YFV.M31/41^ had a significant impact on cell metabolic activity as compared to control sGFP (Figure 3A). A comparable reduction was observed with sGFP^ZIKV.M-31/41^ indicating that ZIKV M oligopeptide had an effect on the metabolic activity of Huh7 cells. To determine whether the sGFP^ZIKV.M-31/41^ construct had the ability to trigger apoptosis in Huh7 cells, caspase activation was assessed by measuring the enzymatic activity of caspases 3 and 7 which are two key effectors of the apoptosis signaling pathway (Figure 3B). Using a caspase-3/7 assay kit, we found a 2.5-fold increase in caspase enzymatic activity in Huh7 cells expressing GFP^DENV-2.M-31/41^ and GFP^YFV.M-31/41^, consistent with their ability to trigger apoptosis in human hepatoma cells [6]. A comparable caspase-3/7 enzymatic activity was detected in Huh7 cells expressing the sGFP^ZIKV.M-31/41^ construct (Figure 3B). Like DENV-2 and YFV, the residues M-31/41 of ZIKV strain BeH819015 had apoptosis-inducing ability in hepatoma cells through caspase-3/7 activation.

**Figure 4:**
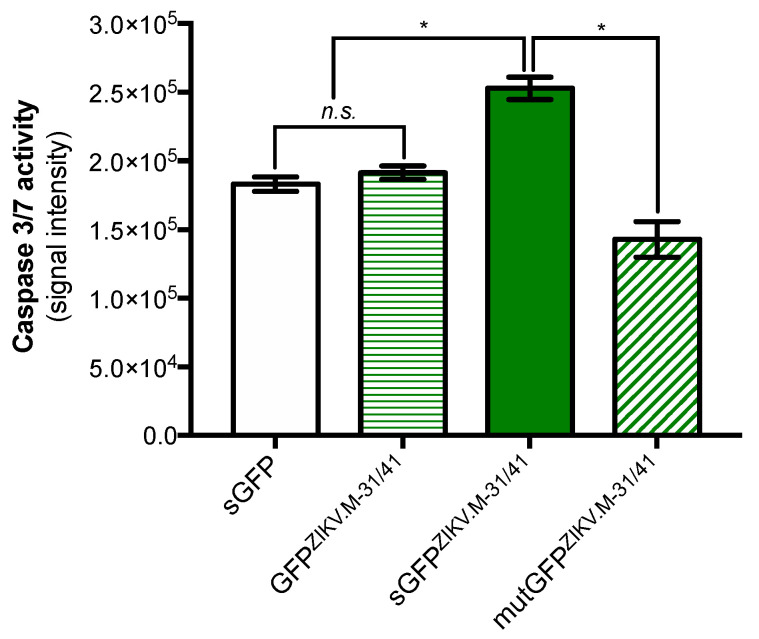
Caspase-associated apoptosis activation requires the signal peptide of sGFP^ZIKV.M-31/41^ and involves the residues M-E33/W35/R38. Huh7 cells were transfected 24 h with plasmids expressing sGFP^ZIKV.M-31/41^ or its mutants lacking a signal peptide (GFP^ZIKV.M-31/41^) or bearing the Ala residues at positions M-33/35/38 (mutGFP^ZIKV.M-31/41^). The sGFP plasmid construct served as a control. Caspase 3/7 enzymatic activity was measured using a caspase-3/7 assay kit, and the O.D. values were expressed as signal intensity. The results are the mean (±SEM) of three independent experiments. Statistical analysis for comparing sGFP-M constructs with sGFP was performed and noted (* *p* < 0.0001; *n.s.*: nonsignificant).

It has been demonstrated that intracellular trafficking of DENV M ectodomain is essential for apoptosis induction [6]. To evaluate whether the same prerequisite exists for ZIKV M oligopeptide, the signal sequence for ER-targeting has been removed from the sGFP^ZIKV.M-31/41^ construct leading to a GFP^ZIKV.M-31/41^ mutant (Figure 1B). The level of caspase-3/7 enzymatic activity was not significantly different in Huh7 cells expressing the GFP^ZIKV.M-31/41^ mutant or the sGFP construct lacking in residues M-31/41 (Figure 4). Thus, the triggering of apoptosis in Huh7 cells involves a transport of soluble GFP^ZIKV.M-31/41^ in the secretory pathway.

We next assessed the possibility of generating a mutant of sGFP^ZIKV.M-31/41^ which had lost its death-promoting activity. It is of note that the residues E-33/35/38 are identical among the M proteins of ZIKV, DENV-2 and YFV (Figure 1A). Moreover, the residue Tryp at position M-35 is strictly conserved among flavivirus M sequences suggesting an important role in apoptosis mediated by flavivirus M ectodomain. By direct mutagenesis, three amino-acid substitutions M-E33A/W35A/R38A were introduced into a sGFP^ZIKV.M-31/41^ construct leading to a mutGFP^ZIKV.M-31/41^ mutant (Figure 1B). As shown in Figure 4, there was a comparable increase in caspase-3/7 enzymatic activity in Huh7 cells expressing mutGFP^ZIKV.M-31/41^ and sGFP control. Thus, introduction of Ala residues at three positions M-33/35/38 dramatically reduced the capacity of sGFP^ZIKV.M-31/41^ to trigger apoptosis suggesting a critical role for M-E33/W35/R38 in the death-promoting activity of the C-terminal amino-acids of ZIKV M ectodomain.

### 2.2. ZIKV M Oligopeptide Confers Death-Promoting Capability to MPF

**Figure 5:**
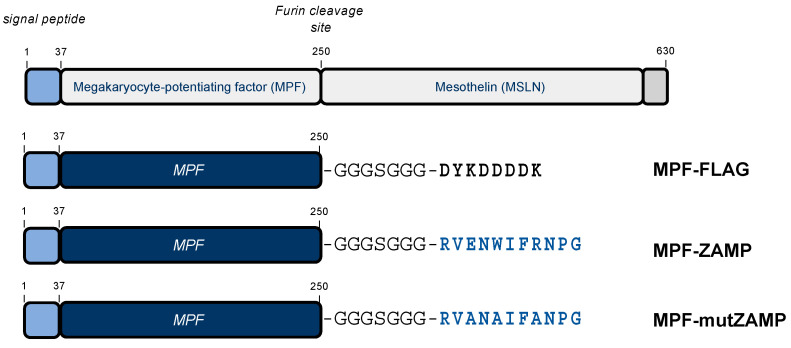
Schematic representation of MPF-ZAMP constructs. At the top, the organization of 69-kDa preprotein mesothelin (MSLN) that is processed into MPF (megakaryocyte-potentiating factor) and membrane-anchored MSLN by furin protease. The signal peptide of MSLN (residues 1/37) is indicated as a blue box. At the bottom, the MPF constructs with at the C-terminus, a Gly−Ser spacer followed by a FLAG epitope or ZIKV M oligopeptide ZAMP. The MPF-mutZAMP construct was made by replacing the three residues M-E31/W35/538 of ZAMP with alanine.

We wondered whether association of ZIKV residues M-31/41 to a human tumor-associated antigen could trigger caspase-associated apoptosis. Mesothelin (MSLN, 630 amino-acid residues) is a tumor-associated antigen which is overexpressed in many human cancers [8]. The megakaryocyte-potentiating factor (MPF, 250 amino-acid residues) is a N-terminal cleavage product from preprotein MSLN [9] (Figure 5). As a human tumor antigen, soluble MPF is released into the circulation serving as a biomarker for the prognosis of lung cancers. The oligopeptide RVENWIFRNPG (hereafter referred to as ZAMP for Zika Apoptosis M Peptide) representing the ZIKV M-31/41 residues was joined to the C-terminus of MPF (Figure 5). The gene coding for human MPF ended by a glycine−serine spacer and ZAMP was inserted into pcDNA3 plasmid. The authentic signal peptide of MPF was used for translocation of MPF-ZAMP into the secretory pathway. A recombinant MPF protein tagged with a FLAG epitope in place of ZAMP was used as a control (Figure 5). Given that the death-promoting activity of mutZAMP had been lost due to mutations on residues M-33/35/28 (Figure 4), a MPF-mutZAMP mutant was made by replacing the residues M-E33/W35/R38 of ZAMP with alanine (Figure 5).

**Figure 6:**
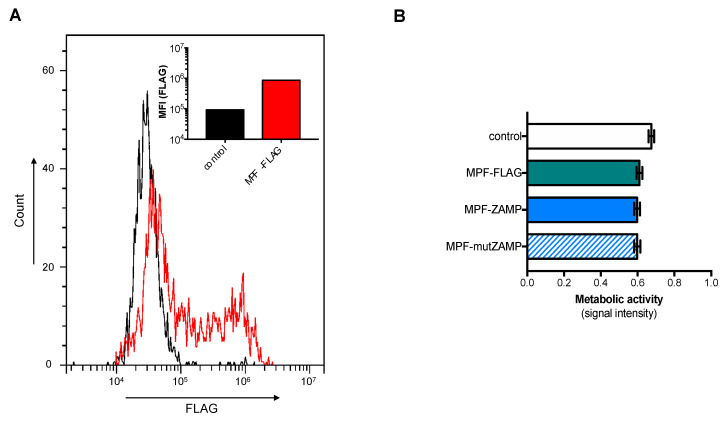
ZAMP associated to MPF has no effect on HEK-293T cells. Cells were transfected 24 h (**A**) or 48 h (**B**) with plasmids expressing MPF-FLAG, MPF-ZAMP, MPF-mutZAMP, or mock-transfected (control). In (**A**), FACS analysis was performed on cells expressing MPF-FLAG using anti-FLAG antibody. The percentage and the mean of fluorescence intensity (MFI) of positive cells are shown. In (**B**), the level of metabolic activity was measured using an MTT assay and expressed as signal intensity (O.D.). The results are the mean (±SEM) of four independent assays. Statistical analysis for comparing MPF constructs with MPF-FLAG was performed and nonstatistical differences were observed.

To validate the expression of our recombinant MPF in human cells, human embryonic kidney HEK-293T cells were transfected with a plasmid expressing MPF-FLAG protein. FACS analysis detected a high expression level of MPF-FLAG in HEK-293T cells using anti-FLAG antibody (Figure 6A). The effects of MPF-ZAMP expression on cell viability were examined by measuring MTT activity. At 48 h post-transfection, there was no change in metabolic activity of HEK-293T cells expressing MPF-ZAMP or MPF-mutZAMP as compared to MPF-FLAG (Figure 6B). Thus, expression of a recombinant MPF bearing ZAMP or its defective mutant had no effect on the viability of HEK-293T cells. The absence of sensitivity of HEK-293T cells to ZAMP expression is consistent with a previous report on a lack of proapoptotic activity of DENV ApoptoM in these cells [6].

**Figure 7:**
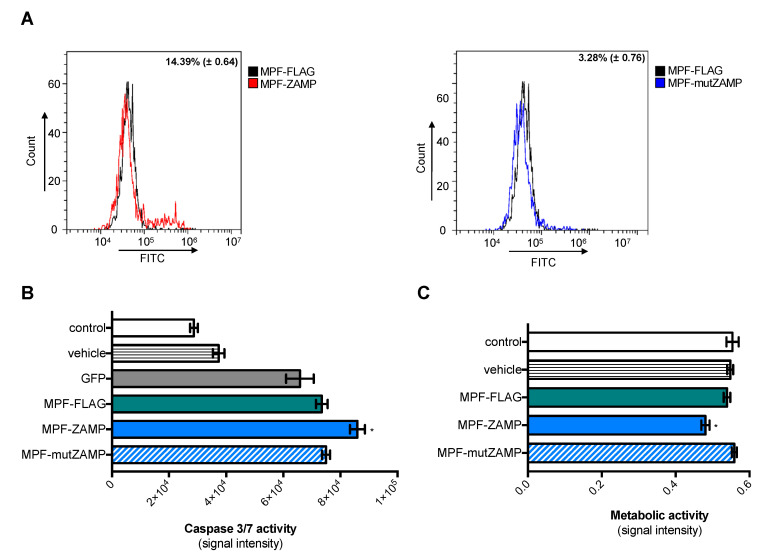
Expression of MPF-ZAMP triggers apoptosis in A549 cells. A549 cells were transfected 24 h with plasmids expressing MPF-FLAG, MPF-ZAMP, MPF-mutZAMP or GFP or transfectant alone (vehicle) or mock-transfected (control). In (**A**), A549 cells were transfected with plasmids expressing MPF-ZAMP or MPF-mutZAMP. Cells in apoptotic state were quantified by staining with FITC-labeled Annexin V. The percentage of Annexin V-positive cells was determined by FACS analysis. The results are the mean (±SEM) of two independent assays. In **(B**), caspase 3/7 enzymatic activity was measured using a caspase-3/7 assay kit, and the O.D. values were expressed as signal intensity. The results are the mean (±SEM) of four independent assays. Statistical analysis for comparing MPF constructs with GFP was performed and noted (* *p* < 0.001); any nonstatistically significant comparisons are omitted. In (**C**), the level of metabolic activity was measured using an MTT assay and expressed as signal intensity (O.D.). The results are the mean (±SEM) of three independent assays. Statistical analysis for comparing MPF constructs with MPF-FLAG was performed and noted (* *p* < 0.001); differences that were not statistically significant are omitted.

Given that MPF is a biomarker of lung cancers [10], pulmonary adenocarcinoma A549 cells were tested with plasmids expressing MPF-ZAMP proteins. FACS analysis using anti-FLAG antibody showed that expression of the MPF-FLAG protein was efficient in A549 cells as observed with HEK-293T cells (Figure S2). The death-promoting activity of MPF-ZAMP in A549 cells was assessed by measuring the rate of early apoptosis using the Annexin V affinity assay, which detects phosphatidylserine (PS) translocation to the outer layer of the cell membrane. At 24 h post-transfection, expression of MPF-ZAMP resulted in a significant increase in the rate of Annexin V-positive A549 cells by at least 14% as compared to control MPF-FLAG (Figure 7A). No Annexin V-positive A549 cells were detected with MPF-mutZAMP mutant.

We next evaluated whether expression of MPF-ZAMP induces caspase activation in A549 cells. A GFP plasmid construct was used as a control of caspase activity in transfected cells. At 24 h post-transfection, a comparable level of caspase-3/7 enzymatic activity was detected in A549 cells expressing MPF-FLAG or GFP. The level of caspase-3/7 enzymatic activity was significantly higher in A549 cells expressing MPF-ZAMP compared to MPF-FLAG (Figure 7B). However, there was no difference in caspase-3/7 activity between MPF-mutZAMP and MPF-FLAG (or GFP) in A549 cells consistent with the fact that Ala mutant of ZAMP has no ability to trigger apoptosis. We examined whether the induction of apoptosis in A549 cells expressing MPF-ZAMP was associated with an impact on cell metabolism activity (Figure 7C). Using a MTT assay, we showed that expression of MPF-ZAMP but not MPF-FLAG or MPF-mutZAMP significantly reduced the level of metabolic activity in A549 cells. Taken together, these results showed that ZAMP has the ability to confer death-promoting capability to MPF.

**Figure 8:**
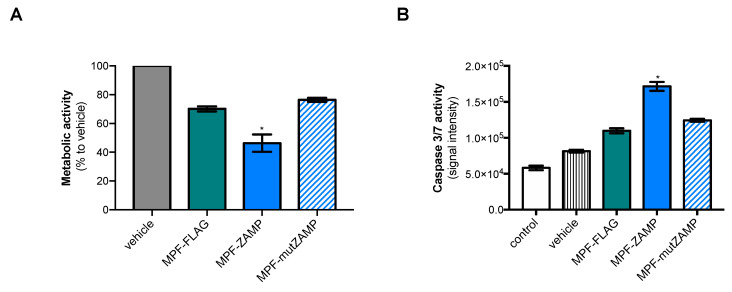
Expression of MPF-ZAMP triggers apoptosis in Huh7 cells. Huh7 cells were transfected with plasmids encoding MPF-FLAG, MPF-ZAMP, MPF-mutZAMP or transfectant alone (vehicle) or mock-transfected (control). In (**A**), the level of metabolic activity in Huh7 cells was determined at 18 h post-transfection using an MTT assay. The results were expressed as a percentage of cell metabolic activity in each assay relative to that in the vehicle and the results were the mean (±SEM) of four independent assays. Statistical analysis for comparing MPF constructs with MP-FLAG was performed and noted (* *p* < 0.001); any nonstatistically significant comparisons were omitted. In (**B**), caspase 3/7 enzymatic activity was measured at 18 h post-transfection using a caspase-3/7 assay kit, and the O.D. values were expressed as signal intensity. The results were the mean (±SEM) of six independent assays. Statistical analysis for comparing MPF constructs with MPF-FLAG was performed and noted (* *p* < 0.0001); any nonstatistically significant comparisons were omitted.

We wondered whether MPF-ZAMP could trigger apoptosis in human hepatoma cells such as Huh7 cells. A MTT assay showed that expression of MPF-ZAMP resulted in a dramatic loss of metabolic activity in Huh7 cells at 24 h post-transfection. Consequently, the impact of MPF-ZAMP expression on the level of cell metabolism activity was examined at 18 h post-transfection. At this timepoint of transfection, the level of metabolic activity was significantly reduced in Huh7 cells expressing MPF-ZAMP as compared to MPF-FLAG or MPF-mutZAMP (Figure 8A). The effect of MPF-ZAMP on cellular metabolism coincided with a high level of caspase-3/7 enzymatic activity in Huh7 cells (Figure 8B). At 24 h post-transfection, a comparable level of caspase-3/7 activation was detected in Huh7 cells expressing MPF-mutZAMP or MPF-FLAG. There was a lack of proapoptotic activity for the ZAMP mutant bearing the Ala mutations (Figure S3). We can conclude that expression of MPF-ZAMP induces caspase-associated apoptosis activation in Huh7 cells as it has been observed with A549 cells.

## 3. Discussion

Flaviviruses expose on their surface the structural proteins E and M, the latter being cleaved from its glycosylated precursor form prM into immature virus particles during their exocytic transit towards extracellular compartments [11]. The mature M protein comprises a 40-residue-long ectodomain followed by a membrane anchoring region composed of two transmembrane domains. Little is still known about the role of small integral membrane M protein in the biology and pathogenicity of flaviviruses. A role has been evoked for M in flavivirus assembly [12,13,14,15]. Recently, a channel activity has been designated to ZIKV M protein promoting virus entry into the host-cell [16]. Historically, we identified a role for the M ectodomain in apoptotic cell death triggered by DENV and YFV [6]. It has been found that proapoptotic activity of the DENV M ectodomain essentially relates to its last C-terminal amino-acid residues which compose a viral apoptosis inducer named ApoptoM [6]. Expression of ApoptoM results in mitochondrial membrane permeabilization leading to caspase-3 activation [6,17].

ZIKV can trigger apoptotic cell death in vitro as well as in vivo [18,19,20,21]. The structural proteins prM and E have been associated with apoptosis mediated by ZIKV [22]. In the present study, we demonstrated that the last C-terminal residues M-31/41 of the ZIKV M ectodomain from Brazilian viral strain BeH819015 have death-promoting activity through caspase-3/7 activation. To our knowledge, this is the first time that a proapoptotic activity has been assigned to the ZIKV M oligopeptide RVENWIFRNPG for which the acronym ZAMP (for Zika Apoptosis M Peptide) has been proposed. We noted that the ZAMP sequence shares at least 60% of its identity with the counterpart from DENV-2 but less than 30% with YFV, despite the fact that the death-promoting activity of the last C-terminal residues of the M ectodomain is conserved among the three flaviviruses. We identified the residues M-33/35/38 as playing a key role in the proapoptotic activity of ZAMP. Noteworthy, the residue Tryp to position M-35 is strictly conserved among flavivirus M proteins. Although it is highly likely that ZIKV cannot tolerate a change of the three residues M-33/35/38 together, it would be of great interest to assess the contribution of each the three residues in relation to the death-promoting activity of ZIKV. We reported that the amino-acid substitution M-L36F which differentiates wild-type YFV to live attenuated YFV vaccine strains abolished the capacity of the M ectodomain to trigger apoptosis [6]. The mutation M-L36F has been also involved in a loss of neurovirulence for Japanese encephalitis virus, a close relative to ZIKV [23]. It will be of great interest to investigate the role of residue M-36 in the pathogenic properties of ZIKV. Such a study could be beneficial for the further development of live attenuated strains as Zika vaccine candidates [24].

The apoptosis signaling pathways involved in ZAMP-mediated cell death still remain to be investigated. Our previous studies on DENV M ectodomain have shown that the triggering of apoptosis involves the trafficking of viral sequences into the secretory pathway [6]. We showed that ZAMP-mediated apoptosis requires the translocation of the M oligopeptide into the secretory pathway. Flavivirus infection causes a consistent increase in the amount of the proapoptotic Bax [25,26]. A dysregulation of MCL-1 expression associated with the inhibition of antiapoptotic BCL-XL protein as well as caspase 8 activation contribute to apoptosis mediated by flaviviruses [27]. In the ER, Sphingosine Kinase SPHK2 can generate proapoptotic ceramides through the production of sphingosine 1-phosphate which acts as an intracellular signaling molecule. Interestingly, SPHK2 has been shown to interact with DENV proteins leading to apoptotic cell death [28]. How this relates to ZAMP will have to be addressed in the future.

In the present study, we demonstrated that ZAMP association with MPF which relates to tumor-associated antigen mesothelin can trigger apoptosis in hepatoma and pulmonary adenocarcinoma cells. Expression of MPF with ZAMP protein results in PS translocation to the outer leaflet of the plasma membrane and caspase-3/7 activation leading to a loss of cell metabolic activity without affecting cell membrane permeability. A lack of apoptosis was observed in HEK-293T cells expressing ZAMP as it has been already reported with the proapoptotic M sequence of DENV-2 [6]. Transformed HEK-293T cells are defective in terms of p53 protein activation [29] that has been described to be the central component in the complex network of apoptosis signaling pathways [30]. The fact that ZAMP induces caspase-3/7 activation in A549 cells having a functional p53 protein [31] as well as p53-deficient Huh7 cells [32] do not sustain a major role for p53-dependent signaling pathway in apoptosis mediated by ZAMP. The megakaryocyte-potentiating factor is part of the tumor-associated antigen (TAA) mesothelin. Over the past several decades, several useful TAAs have been identified and characterized for their biochemical properties. TAAs represent a group of normal nonmutant molecules and can be subdivided into four major categories according to expression pattern: (1) cancer-testis antigens such as MAGE-1 are expressed in a wide range of different cancers including melanoma, blade cancer and neuroblastoma [33]; (2) Differentiation antigens including tyrosinase TRP-1 are expressed in differentiation stage-dependent and tissue-specific manners [34,35]; (3) oncofetal antigens including carcinoembryonic antigen (CEA) are found in embryonic and fetal tissues as well as certain cancers [36,37]; (4) overexpressed antigens such as mesothelin that are normal proteins whose expression is upregulated in cancer cells [9].

Recently, ZIKV has gained attention in the oncolytic virotherapy field as a potential oncolytic virus due to its neurotropism and its efficacy against glioblastoma multiforme and aggressive metastatic forms of human CNS tumors [38,39,40]. As aforementioned, the association of ZAMP to MPF was efficient for the triggering of apoptosis in different types of tumor cells. The ability of ZAMP to confer proapoptotic capability to TAA could open important perspectives for such a ZIKV M oligopeptide in cancer therapy strategies through tumor cell death and immune recognition of the TAA by antigen-presenting cells.

## 4. Materials and Methods

### 4.1. Cell Lines and Antibodies

Huh7 cells (a generous gift from Dr N. Jouvenet, Institut Pasteur, Paris, France), A549 cells (ATCC, CCL-185) and HEK-293T cells (ATCC, CRL-1573) were cultured in Minimum Essential Media (MEM) supplemented with 10% heat-inactivated fetal bovine serum (FBS) and nonessential amino acids. The mouse anti-DDDDK tag mAb (anti-FLAG_tag_ antibody) and goat anti-mouse immunoglobulin-horseradish peroxidase (HRP) conjugated secondary antibody were purchased from Abcam (Cambridge, UK).

### 4.2. Vector Plasmids Expressing GFP and MPF Constructs

A synthetic gene coding for the reporter gene GFP preceded by the last C-terminal amino-acids of ZIKV C protein (Genbank reference number KU365778) which which compose the prM signal peptide and followed by a FLAG epitope was synthesized and then inserted into *Nhe* I and *Not* I restriction sites of vector plasmid pcDNA-3.1 by Genecust (Boynes, France). The GFP constructs ended by a Gly-Ser spacer followed by the residues M-31/41 of DENV-2 strain RUJul (Genbank reference number MN272404), YF-17D vaccine strain (Genbank reference number X03700), or epidemic ZIKV strain BeH819015 (Genbank reference number KU365778) were synthesized and then inserted into *Nhe* I and *Not* I restriction sites of pcDNA-3.1 by Genecust (Boynes, France). A synthetic gene coding for the soluble MPF fragment (residues MSLN-37/250) from MSLN protein (Genbank reference number NM_001177355.3) preceded by its signal peptide was synthetized and inserted into *Nhe* I and *Not* I restriction sites of pcDNA-3.1 by Genecust (Boynes, France). The MPF constructs ending in a FLAG epitope or the ZIKV residues M-31/41 were synthetized and inserted into *Nhe* I and *Not* I restriction sites of pcDNA-3.1 by Genecust (Boynes, France). The production of endotoxin-free plasmids, the quantification of plasmid DNA, and the sequencing of plasmids were performed by Genecust (Boynes, France). Cells were transfected with GFP or MPF constructs using Lipofectamine 3000 (Thermo Fisher Scientific, Les Ulis, France) according to the manufacturer’s instructions.

### 4.3. Flow Cytometry Analysis

Huh7 cells (1.5 × 10^5^), HEK-293T cells (1 × 10^5^) or A549 cells (1 × 10^5^) were fixed with 3.7% paraformaldehyde (PFA) in phosphate buffered saline (PBS) for 10 min and then observed for GFP expression. For the detection of FLAG epitope, cells were stained with anti-FLAG antibody (dilution 1:2000) for 1 h at room temperature. Goat anti-mouse Alexa Fluor 488 IgG antibody was used as a secondary antibody. For each assay, 10^4^ cells were analyzed by flow cytometry (CytoFLEX, Beckman Coulter, Brea, CA, USA) using CytExpert software (version 2.1.0.92, Beckman Coulter, Brea, CA, USA).

### 4.4. Lactate Dehydrogenase Assay

Huh7 cells were seeded in a 12-well culture plate at a density of 1.5 × 10^5^ cells per well. Cell damage was evaluated on supernatants of transfected-cells measuring lactate dehydogenase (LDH) release using Cytotox 96 nonradioactive cytotoxicity assay (Promega, Charbonnières-les-bains, France) according to manufacturer’s instructions. Absorbance of converted dye was measured at 490 nm with a background subtraction at 690 nm using a microplate reader Tecan (Tecan Trading AG, Männedorf, Switzerland).

### 4.5. MTT Assay

Huh7 cells (2 × 10^4^), A549 cells (2 × 10^4^) or HEK-293T cells (2 × 10^4^) were cultured in a 96-well culture plate. Cell monolayers were rinsed with PBS 1× and incubated with culture growth medium mixed with 5 mg mL^−1^ MTT (3-[4,5-dimethylthiazol-2-yl]-2,5-diphenyltetrazolium bromide) solution for 1 h at 37 °C. MTT medium was removed and the formazan crystals were solubilized with dimethyl sulfoxide (DMSO). Absorbance was measured at 570 nm with a background subtraction at 690 nm.

### 4.6. Caspase 3/7 Enzymatic Activity

Huh7 (2 × 10^4^) or A549 (2 × 10^4^) cells were seeded in a 96-well culture plate. Caspase 3/7 enzymatic activity in raw cell lysates was measured using a Caspase Glo 3/7 assay kit (Promega) according to the manufacturer’s protocol. Caspase activity was quantified by luminescence using a FLUOstar Omega Microplate Reader (BMG Labtech, Champigny-sur-Marne, France).

### 4.7. Early Apoptosis Analysis

A549 cells were seeded in a 12-well culture plate at a density of 1 × 10^5^ cells per well. Cells were fixed with 3.7% PFA in PBS for 10 min and stained with the Ca^2+^-dependent phosphatidylserine-binding protein FITC-labelled Annexin V (BioLegend, San Diego, CA, USA). For each assay, 10^4^ stained cells were were analyzed by flow cytometry (CytoFLEX, Beckman Coulter, Brea, CA, USA) using CytExpert software (version 2.1.0.92, Beckman Coulter, Brea, CA, USA).

### 4.8. Statistical Analysis

Statistical analysis for comparison studies was performed using GraphPad Prism software (version 9, GraphPad software, San Diego, CA, USA). Values of independent experiments were analyzed by one-way ANOVA using Dunnett’s multiple comparisons test. Values of *p* < 0.05 were considered statistically significant.

## Supplementary Materials

The following are available online at https://www.mdpi.com/1422-0067/21/24/9578/s1, Figure S1: Expression of sGFP constructs in transfected Huh7 cells. Figure S2: Expression of MPF-FLAG construct in A549 cells; Figure S3: Caspase-3/7 enzymatic activity in A549 cells expressing MPF-mutZAMP mutant.

## Abbreviations

ApoptoM	Dengue M apoptotic oligopeptide
C	Capsid protein
CNS	Central nervous system
DENV	Dengue virus
DENV-2	Dengue virus type 2
DMSO	Dimethyl sulfoxide
E	Envelope protein
ectoM	M ectodomain
ER	Endoplasmic reticulum
FBS	Fetal bovine serum
GFP	Green fluorescent protein
LDH	Lactate dehydrogenase
M	Membrane protein
MEM	Minimum Essential Media
MFI	Mean fluorescence intensity
MPF	Megakaryocyte-potentiating factor
MSLN	Mesothelin
NS	Nonstructural protein
PBS	Phosphate buffered saline
PFA	Paraformaldehyde
prM	Intracellular precursor of membrane protein
PS	Phosphatidylserine
sGFP	Soluble green fluorescent protein
SP	Signal peptide
SPHK2	Sphingosine kinase 2
TAA	Tumor-associated antigen
WNV	West Nile virus
YFV	Yellow Fever virus
ZAMP	Zika Apoptosis M Peptide
ZIKV	Zika virus
