# Supplementary material for: Zika M Oligopeptide ZAMP Confers Cell Death-Promoting Capability to a Soluble Tumor-Associated Antigen through Caspase-3/7 Activation
Source: Int J Mol Sci. 2020 Dec 16;21(24):9578. doi: 10.3390/ijms21249578 (PMC7765671; doi:10.3390/ijms21249578)
Supplement: Supplementary file 1 [file ijms-21-09578-s001.pdf]

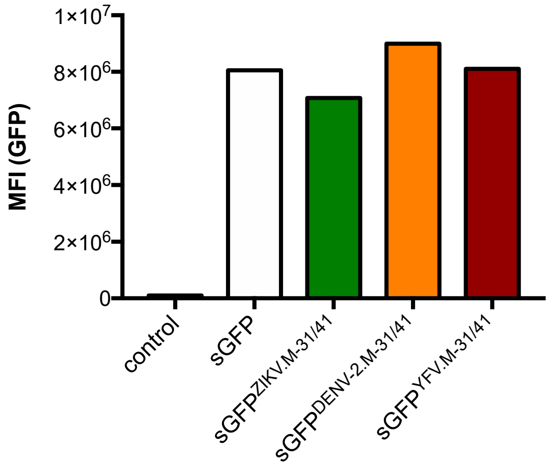

Figure S1. Expression of sGFP constructs in transfected Huh7 cells. Huh7 cells were transfected 24 h with plasmids expressing sGFP, different sGFP-M oligopeptide constructs, or mock-transfected (control). The GFP-positive cells were detected by FACS analysis and their MFI are shown.

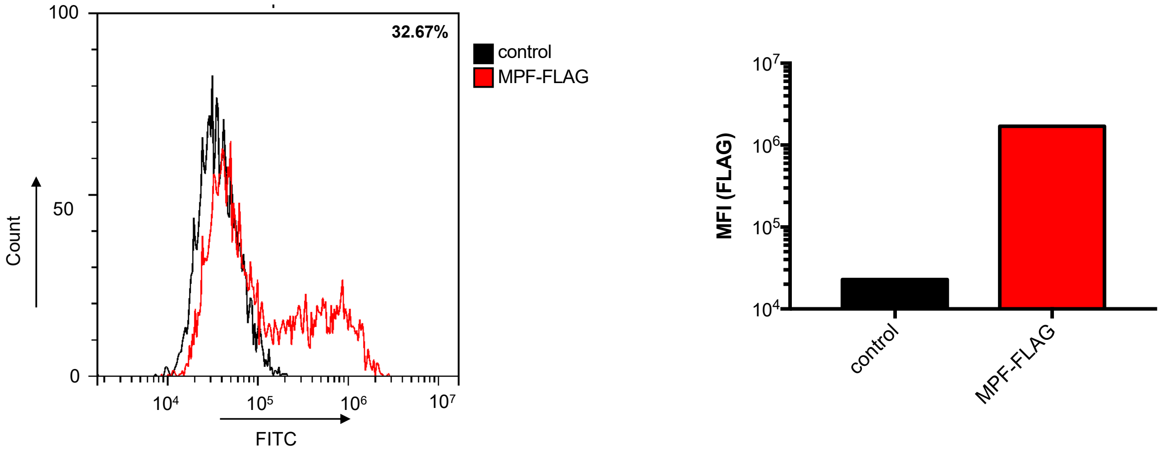

Figure S2. Expression of MPF-FLAG construct in A549 cells. A549 cells were transfected 24 h with plasmids encoding MPF-FLAG construct or mock-transfected (control). Cells were incubated with mouse anti-FLAG antibody as primary antibody and Alexa Fluor 488 anti-mouse IgG antibody as secondary antibody. The percentages of positive cells for MPF-FLAG expression (left) and their MFI (right) were determined by FACS analysis.

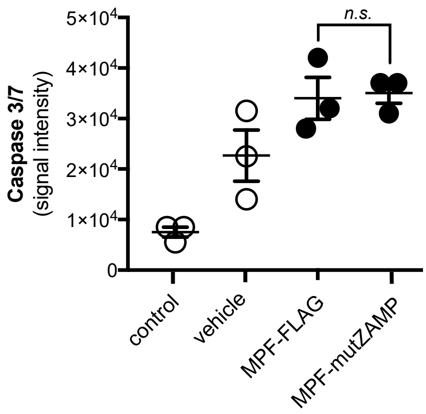

Figure S3. Caspase-3/7 enzymatic activity in A549 cells expressing MPF-mutZAMP mutant. A549 cells were transfected 24 h with plasmids expressing MPF-FLAG, MPF-mutZAMP or transfectant alone (vehicle) or mock-transfected (control). Using a caspase-3/7 assay kit, caspase 3/7 enzymatic activity was determined and the O.D. values were expressed as signal intensity. The results are the mean (±SEM) of four independent assays. Statistical analysis for comparison of MPF-FLAG with MPF-mutZAMP was performed and noted (*n.s.*: non-significant).
